# Bladder Endometriosis: Diagnostic, Therapy, and Outcome of a Single-Center Experience

**DOI:** 10.3390/diagnostics15040466

**Published:** 2025-02-14

**Authors:** Elvin Piriyev, Sven Schiermeier, Thomas Römer

**Affiliations:** 1 Chair of Gynecology and Obstetrics, University Witten-Herdecke, 58455 Witten, Germany; 2 Department of Obstetrics and Gynecology, Academic Hospital Cologne Weyertal, University of Cologne, Weyertal 76, 50931 Cologne, Germany; 3 Chair of Gynecology and Obstetrics, University of Cologne, 50923 Koln, Germany

**Keywords:** bladder endometriosis, DIE, dysuria, hematuria, bladder shaving, partial bladder resection, laparoscopy

## Abstract

**Background/Objectives:** Endometriosis is a benign condition affecting up to 10% of women at reproductive age. The urinary tract is affected in 0.3–12.0% of women with endometriosis and in 19.0–53.0% of women with deep infiltrating endometriosis. The bladder is the most commonly affected organ in the urinary tract. Bladder endometriosis is defined by the presence of endometriosis lesions in the detrusor muscle, with partial or complete thickness involvement. **Methods:** This was a retrospective study. The study analyzed surgical reports of 11,714 patients who underwent endometriosis laparoscopy, and included only 42 patients with bladder endometriosis. **Results:** We found that 0.35% of patients with endometriosis had bladder endometriosis. In total, 29 patients underwent phone follow-up. In total, 26 patients (90%) reported a general improvement in their symptoms (e.g., improving the dysmenorrhea, lower abdominal pain), with a 100% improvement in their dysuria. Only two patients (7%) reported no change in symptoms (dysmenorrhea and dyspareunia). **Conclusions:** Gynecologists can perform laparoscopic surgical treatment of bladder endometriosis in most cases. If ureteroneocystostomy is required or the localization of the endometriosis nodule is unfavorable, an intervention by an interdisciplinary team is recommended. Both laparoscopic partial bladder resection and shaving can be considered effective methods with low complication risk. This surgical approach requires excellent laparoscopic skills.

## 1. Introduction

Endometriosis is defined as a disease characterized by the presence of endometrium-like epithelium and/or stroma outside the endometrium and myometrium, usually with an associated inflammatory process. The pathogenesis of endometriosis is largely unknown. Endometriosis is likely a hereditary chronic condition with an unidentified route of inheritance [1,2]. The epidemiology of endometriosis is poorly known, and the current evidence does not enable definite conclusions [3]. For investigation of the true prevalence, a surgical diagnosis is required [4]. However, estimates suggest that up to 10% of women of reproductive age—or 190 million women worldwide—suffer from endometriosis. Women who are asymptomatic have a prevalence of 2 to 11%. The prevalence of endometriosis in infertile women is 25–50% [5]. Hospitalized women with pelvic pain have a prevalence of endometriosis of up to 21%. The prevalence of endometriosis among symptomatic adolescents varies from 49% for those with chronic pelvic pain to 75% for those with pain that is unresponsive to medical treatment [6]. We can divide endometriosis into three main types: superficial peritoneal endometriosis, ovarian endometriosis, and deep infiltrating endometriosis [7]. If the infiltration of the peritoneum is more than 5 mm, it is defined as deep infiltrated endometriosis [8]. This form of endometriosis is considered the most severe. In total, 1% of women in reproductive age have deep infiltrating endometriosis [7]. One of the most common organ systems affected by endometriosis is the urinary tract. The prevalence ranges from 0.3 to 12% in all women with endometriosis and 19–53% in women with deep infiltrated endometriosis [9,10,11]. The bladder is the most common localization of urinary tract endometriosis, with a prevalence of up to 80% of cases, followed by the ureters, with an incidence of 9–23% [9,12]. Bladder endometriosis is defined by the presence of endometriosis lesions in the detrusor muscle, with partial or complete thickness involvement [13]. However, in the majority of cases, the mucosa is not involved [14]. If endometriosis lesions are located in the bladder peritoneum without infiltrating the bladder muscle, they should be classified as peritoneal endometriosis rather than bladder endometriosis [13]. Bladder endometriosis can cause a variety of typical symptoms, such as suprapubic pain with polyuria (41%), dysuria (21%), hematuria (up to 19%), and recurrent urinary tract infections [15,16]. However, patients with endometriosis of the urinary tract can also have common symptoms, such as dysmenorrhea, dyspareunia, dyschezia, and non-cyclical pelvic pain [15]. The #ENZIAN classification assigns “FB” to bladder endometriosis [17]. Two theories can explain the origin of bladder endometriosis: primary or spontaneous and secondary. The primary theory explains two possible ways to develop bladder endometriosis. The first is the implantation of transtubally regurgitated menstrual endometrium on peritoneal surfaces and the development of adhesions [16,18]. The second is the metaplasia of anterior adenomyosis [19,20]. The secondary theory says that bladder endometriosis occurs when the bladder is injured by uterine surgeries such as hysterectomy, fibroid enucleation, or cesarean section and endometrial cells get into the bladder [21]. The purpose of this study is to demonstrate the results of our center’s diagnosis and treatment of bladder endometriosis, a condition that, despite its rarity, warrants accurate diagnosis and particularly effective therapeutic treatment.

## 2. Materials and Methods

**Figure 1:**
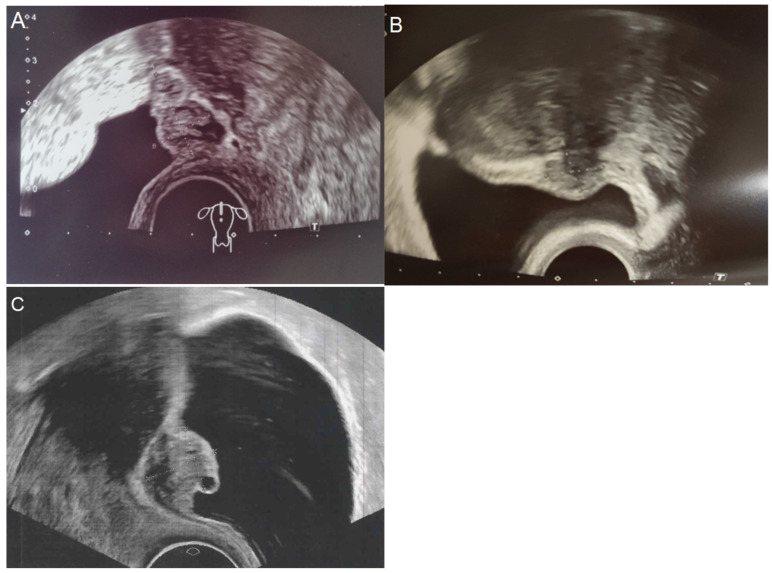
Transvaginal sonography. (**A**) The endometriosis nodule is located on the bladder dome and the bladder base. (**B**) The endometriosis nodule is located on the bladder dome, far from the bladder base. (**C**) The endometriosis nodule is located on the bladder dome, close to the bladder base.

This is a single-center retrospective study. Academic Hospital Weyertal is a Level III Endometriosis Center of Excellence, with up to 1000 endometriosis surgeries annually. We analyzed surgical reports of 11,714 patients who underwent an endometriosis laparoscopy (laparoscopic excision of any type of endometriosis) in our department between January 2014 and December 2024. Only patients with bladder endometriosis (42) were included in this analysis. The most common indications for the surgery were dysmenorrhea and dysuria. We used vaginal sonography to preoperatively assess the size and localization of the endometriosis nodule (Figure 1). In total, 31 cases (74%) (including all cases with full-thickness infiltrated endometriosis) were diagnosed due to transvaginal sonography. The localization of bladder endometriosis was defined as suggested by the IDEA group as follows: bladder dome, bladder base, trigone, and extra-abdominal [22]. We measured the endometriosis nodule’s size using its largest diameter. Additionally, we performed kidney sonography in all cases to rule out hydronephrosis as a potential indicator of ureter obstruction. In our center, we routinely perform cystoscopy in patients with severe dysuria, especially if bladder endometriosis is suspected, by ultrasound. We carried out a simultaneous cystoscopy to assess the bladder mucosa and measure the exact distance between the endometriosis nodule and ureter orifices. Histological confirmation of resected nodules was mandatory. On the one hand, it enabled exclusion of other diseases like bladder cancer. On the other hand, it allowed for the precise evaluation of the nodule’s size. Depending on the depth of the endometriosis infiltration, we implemented two surgical approaches: laparoscopic partial bladder resection for the full thickness infiltration and shaving if mucosa was not affected. We contacted the patients by phone for the follow-up and questioned them about symptom relief, postoperative bladder dysfunction, recurrence, and reintervention. For the classification of the endometriosis, we used the ASRM and ENZIAN/#ENZIAN classifications [17,23,24]. We conducted statistical analyses using a two-tailed Fisher’s exact test, descriptive statistics, confidence intervals of the mean, and a t-test to compare two means. The data are given as the mean and standard deviations.

### Surgical Approach

**Figure 2:**
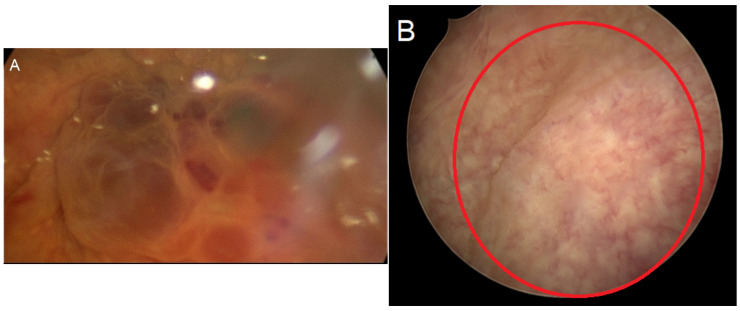
Cystoscopy. (**A**) Mucosa is infiltrated. (**B**) Protrusion of mucosa by the endometriosis nodule, without infiltration.

**Figure 3:**
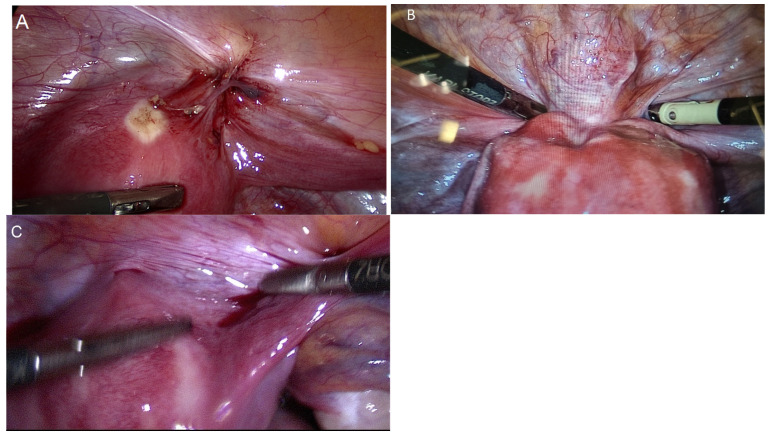
Laparoscopic evaluation. (**A**) Bladder endometriosis lesion and adhesion between the uterus and the bladder. (**B**) Adenomyosis and bladder endometriosis. (**C**) Lateral bladder nodule, right wall endometriosis. Bladder dome is unaltered.

**Figure 4:**
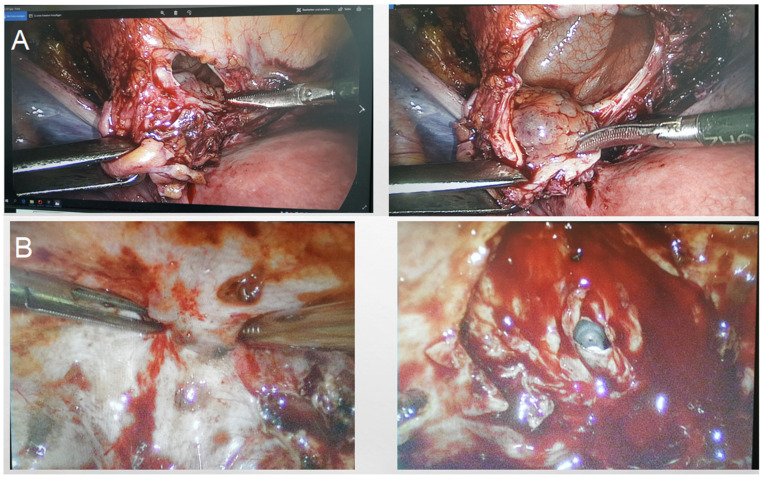
Laparoscopic partial bladder resection. (**A**) Partial bladder resection. (**B**) Shaving. The bladder mucosa is merely opened but not resected.

**Figure 5:**
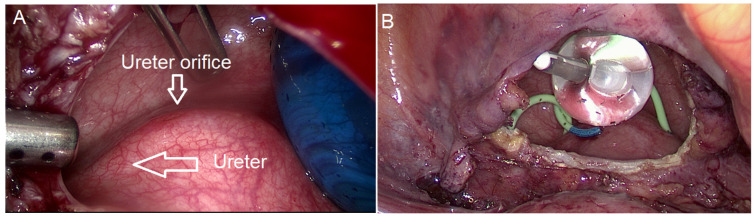
Visualization of the ureters. (**A**) Detection of the ureter and ureter orifice. (**B**) Ureters and D-J stents.

**Figure 6:**
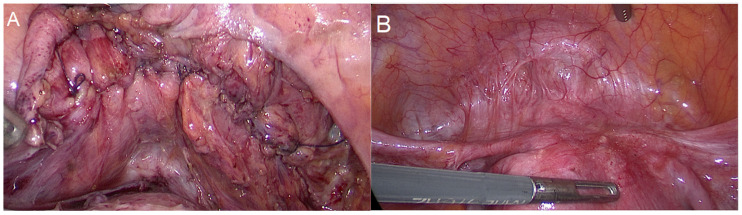
Suture. (**A**) Laparoscopic suture of the bladder after the bladder part resection. (**B**) Six months after the partial bladder resection.

Three experienced laparoscopic surgeons, certified by the German Society for Gynecological Endoscopy at minimal invasive surgery (MIS) Levels II and III, performed all surgeries. We divided the surgical procedure into some sequential steps. The first step was a cystoscopy (Figure 2). During the cystoscopy, bladder mucosa, localization of nodules, and the distance between the nodule and ureters were evaluated. We did not perform transurethral resection in any cases, as we believed that this method alone could not achieve complete resection and also carried a high risk of bladder perforation. The literature also supports our conclusion [25,26,27,28]. The second step was laparoscopic visualization. We evaluated the bladder dome, other localized endometriosis, adhesions between the bladder and uterus, and adenomyosis, particularly if there was a globular appearance of adenomyosis in the uterus (Figure 3). However, the globular appearance is not a direct criterion for adenomyosis and is not present in every patient with the disease. In the third step, we performed adhesiolysis and the necessary dissection. In the fourth step, we performed either partial bladder resection or shaving, depending on the depth of endometriosis infiltration (Figure 4). The partial bladder resection involves opening the bladder to its full thickness in order to completely excise the lesion. This approach has the lowest risk of recurrence [29]. Shaving refers to the complete removal of endometriosis that has infiltrated the bladder muscle without breaching the mucosa [15]. In the case of partial bladder resection, the next step involves evaluating the ureters and their orifices (Figure 5). This step is crucial to prevent ureter obstruction during suturing. The final, sixth, step is suturing (Figure 6). If we performed deep shaving, we sutured the bladder wall to prevent a potential fistula. We sutured the bladder in two layers after partial bladder resection and in one layer after the shaving. We preferred continuous suturing in all cases. Following a deep shaving procedure with suturing and partial bladder resection, the patients received a bladder catheter for a period of 7–10 days.

## 3. Results

### 3.1. Patient Characteristics

This retrospective, single-center study evaluated patients who underwent laparoscopic endometriosis surgery. The analysis included 42 patients with bladder endometriosis in total. In total, 37 out of 42 patients underwent a laparoscopic partial bladder resection. At the time of this analysis, bladder surgery had not taken place in five cases. However, we incorporated these cases into the analysis to assess additional statistical data, including symptoms, adhesions, and endometriosis in other locations. We evaluated 11,714 endometriosis surgeries and found a prevalence of bladder endometriosis of 0.35%. The patients’ ages ranged from 25 to 48 years old. The main symptoms were dysmenorrhea and dysuria, followed by dyspareunia and dyschezia. Even though endometriosis affected the bladder mucosa in 29 cases (detected by cystoscopy), only four patients reported hematuria. Fourteen patients had symptoms such as dysuria, dyspareunia, and low abdominal pain despite hormonal treatment. A total of 25 patients underwent at least one previous surgery, including 30 laparoscopies, 3 cesarean sections, and 6 uterus curettages. Twenty patients underwent at least one laparoscopy due to endometriosis, and two of them underwent a simultaneous hysterectomy. Seven patients gave birth. Table 1 illustrates the main patients’ demographic and clinicopathological characteristics.

### 3.2. Preoperative Management

The preoperative assessment of the bladder endometriosis nodule was performed by vaginal and abdominal sonography. In the past, we regularly used D-J stents. But, later, we started using them only in selected cases, e.g., if the nodule was big, lay close to the trigone, or if a surgery on the ureter (ureteroneocystostomy) was planned. In total, 38% of patients obtained D-J stents (Table 2).

### 3.3. Intraoperative Outcome

In total, 37 patients underwent laparoscopic bladder resection, with 27 undergoing a partial bladder resection and 10 undergoing a shaving procedure. A urologist’s involvement was necessary in four cases. In two cases, a ureteroneocystostomy was planned in advance. We used Vicryl, barbed, and polydioxanone suture (PDS) sutures for the bladder suture. A cystoscopy for evaluating bladder mucosa, endometriosis nodules, as well as the exact distance between the endometriosis nodule and ureter orifices, was performed. The bladder mucosa was completely infiltrated in 23 cases, while in 2 cases, the endometriosis nodule bulged into the mucosa without complete infiltration. However, the partial bladder resection was necessary in 27 cases, since the deep infiltration of mucosa (even when not completely) required the full thickness resection. Histopathological reports verified the size of the endometriosis nodule, which ranged from 1 to 4.5 cm. The majority of patients had a nodule of 2–4 cm. In five cases, the distance between the endometriosis nodule and the trigon was less than 2 cm. None of these patients had an obstruction of the ureter. Out of these five patients (with close localization of the endometriosis nodule to the trigon), three underwent laparoscopic partial bladder resection, whereby two patients were operated on interdisciplinary with a urologist to perform the ureteroneocystostomy.

**Figure 7:**
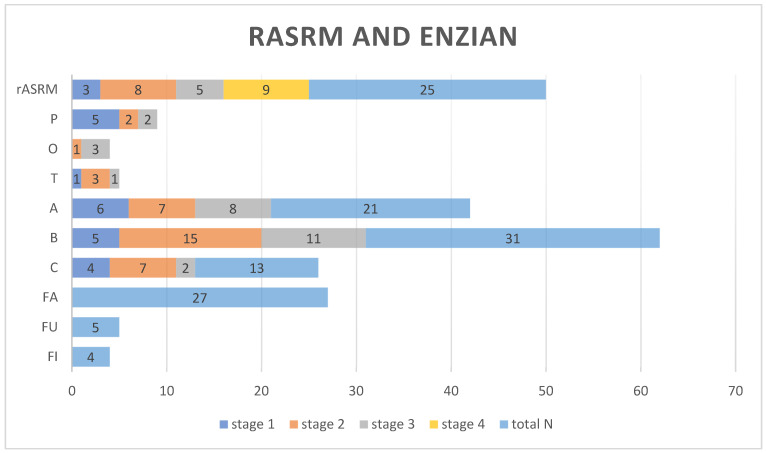
Number of patients relating to rASRM and ENZIAN classification. rASRM—Revised American Society of Reproductive Medicine, classification of endometriosis; ENZIAN, classification of deep infiltrating endometriosis. P—peritoneal endometriosis, O—ovarian endometriosis, T—Adnexal adhesions, A—vagina, rectovaginal space, B—uterosacral ligaments/cardinal ligaments/pelvic sidewall, C—rectum endometriosis, FA—adenomyosis, FU—ureter endometriosis, FI—other intestinal locations (sigmoid colon, small bowel).

During laparoscopy, we determined adhesions, adenomyosis, and endometriosis in other localizations. In total, 20 patients had previous endometriosis laparoscopy. However, the endometriosis in the bladder remained unaffected. For this reason, we assumed that the adhesions between the bladder and uterus were the same. Endometriosis classification was performed using rASRM and ENZIAN/#ENZIAN (Figure 2). Since the #ENZIAN classification was widely integrated into our hospital in 2022, most cases were classified using a combination of rASRM and ENZIAN. Therefore, most endometriomas were included in rASRM and not in “O” in #ENZIAN. Considering the rASRM classification, 21% of patients had stage IV endometriosis. The prevalence of deep infiltrated endometriosis of the vagina, sacrouterine ligaments, and bowel was 50% (21 patients), 74% (31 patients), and 31% (13 patients), respectively. Only one patient had beside the bladder endometriosis “merely” adenomyosis. Table 2 and Figure 7 display the main intraoperative outcomes.

### 3.4. Postoperative Outcomes

Postoperative bleeding occurred in three out of the 27 cases following a partial bladder resection. Two of these patients had 4 cm nodules, whereas one had a 2 cm nodule. In two cases, a re-laparoscopy was required, whereas in one, the bleeding stopped spontaneously. One patient developed ureteral stenosis following a partial bladder resection with a 3.5 cm endometriosis lesion, necessitating urological reintervention. There were no complications following shaving.

Twenty-nine patients underwent phone follow-up. Twenty-six patients (90%) reported an improvement of symptoms in general, and an increase in quality of life. Twenty-two (76%) out of twenty-nine patients had dysuria before the surgery, and all patients reported an improvement of dysuria (100%) after the surgery. Only two patients (7%) reported no change in symptoms (dysmenorrhea and dyspareunia). Two patients (7%) reported bladder dysfunction. However, in one case, the patient reported mild bladder dysfunction, and it did not affect her quality of life. In another case, the patient already had bladder dysfunction prior to surgery, and it did not worsen after the procedure. Two patients (7%) reported mild postoperative pollakiuria. Four patients (with mild bladder dysfunction and pollakiuria) were satisfied with postoperative results owing to a significant improvement in symptoms and increased quality of life. Until follow-up, no patient underwent reintervention due to a recurrence of endometriosis.

## 4. Discussion

Bladder endometriosis is a rare condition. However, if patients have bladder symptoms in the absence of urinary infection, this diagnosis should be considered [16]. The literature estimates the prevalence of urinary tract endometriosis in all patients with endometriosis at 0.3–12%, with the bladder being the most common localization [9,10]. It is to be highlighted that the “true” bladder endometriosis means the involvement of the bladder muscle, with or without infiltration of the mucosa, and not the affection only of the bladder peritoneum or uterovesical fold [13,30]. Khazali et al. evaluated 1160 patients with endometriosis in a very recent study. The study revealed a 6% prevalence of bladder endometriosis [31]. The present study estimated the prevalence of bladder endometriosis to be 0.35%, which is significantly lower than the recent data. However, the authors of this paper believe that the data from the present study would more accurately reflect the true prevalence of bladder endometriosis, for the following reasons. (1) We evaluated a large number of patients (11,714 patients) with endometriosis. (2) All patients underwent a laparoscopy, and endometriosis was confirmed histologically. (3) We investigated all patients with diagnosed endometriosis, regardless of the type (peritoneal, deep infiltrating, or cystic endometriosis).

The pathogenesis of bladder endometriosis is not fully understood, and the complete pathways are not known [2,32,33]. However, through a thorough evaluation of the literature, we found some theories that could explain the origin of the bladder endometriosis. Some authors support the theory of retrograde menstrual bleeding and implantation of endometrial cells on the anterior cul-de-sac. Following implantation, the inflammatory process promotes the formation of adhesions between the bladder and the uterus, leading to the formation of a fibrotic nodule, likely from the vesicovaginal septum, buried beneath the peritoneum [16,18]. Other authors see the cause of bladder endometriosis in the metaplasia of anterior adenomyosis [19,20]. For this reason, they propose a simultaneous, partial resection of the anterior uterine wall during the treatment of bladder endometriosis. Both theories can be deemed primary. The high prevalence of adenomyosis (64%) and adhesions between the bladder and uterus (69%) in our case series could confirm these theories (Table 2). The third theory, known as secondary, posits that the origin of bladder endometriosis can be traced back to damage to the bladder during surgery on the uterus, specifically the opening of the cavity. As a result, endometrial cells might directly affect the bladder wall [21]. Interestingly, only 1 patient out of the 42 included in the present study had no further endometriosis lesions, except for bladder endometriosis and adenomyosis. The medical history of this patient included a cesarean section. However, regarding the secondary theory, due to the absence of evidence, it is crucial to exercise extreme caution when making such claims. These observations indicate that there is still a need for further clarification of the existence of bladder endometriosis entities after prior uterine surgery. All these theories explain the central localization of the bladder endometriosis, but not the lateral lesions. From our point of view, lateral lesions occur as a result of metaplasia of parametrial endometriosis.

Patients with bladder endometriosis can have several symptoms, including but not limited to dysmenorrhea, dyspareunia, dyschezia, and non-cyclical pelvic pain [34]. The most common specific symptoms associated with bladder endometriosis are dysuria and hematuria, although other symptoms of lower urinary tract can occur, including painful bladder filling, urgency, frequency, incontinence, and voiding dysfunction [21,35]. A recent review revealed that patients with bladder endometriosis had rates of dysuria and hematuria of 27.18% and 10.77%, respectively. However, Ceccaroni et al. published a series of 264 patients with bladder endometriosis, revealing a significantly higher rate of dysuria (67.1%) and slightly higher hematuria (18.9%). The rate of dysmenorrhea was 96.1% [36]. Our results are equivalent to the results of Ceccaroni et al.: dysmenorrhea 96.1% vs. 88%, *p* 0.0526; dysuria 67.1% vs. 69%, *p* 0.8609; hematuria 18.9% vs. 9.5%, *p* 0.1900. Interestingly, in our study, although a cystoscopy in 25 cases revealed an endometriosis nodule, indicating mucosal infiltration, only 4 patients reported hematuria. Infiltration of the mucosa may not be the only cause of hematuria; endometriosis cell activity, inflammation, nodule location, and detrusor activity may also contribute. Further studies should investigate this. Furthermore, we did not note any correlation between the size and severity of the symptoms.

The therapy of bladder endometriosis includes medical and surgical options. The evidence supporting medical treatment for bladder endometriosis aligns with general endometriosis management guidelines, suggesting that such treatment can improve symptoms associated with endometriosis [37]. Progestogens and combined oral contraceptives should be considered the first option for hormonal treatment [38,39,40]. However, the literature on hormonal therapy for bladder endometriosis is limited [30]. Whereas some authors showed good results of hormonal therapy [21,41,42], other authors reported that the bladder endometriosis nodule itself and associated symptoms may respond suboptimally to hormone therapy [39,43]. Another point is that medical therapy is effective in temporarily suppressing, but not curing, bladder endometriosis [21,39]. Taking all facts into account, hormonal treatment should be offered as an option to all patients with bladder endometriosis without current pregnancy intention even if surgical management is ultimately planned [21,44]. If patients opt for hormone therapy, it is crucial to educate them about the long-term nature of the treatment and the potential for bladder endometriosis to worsen, necessitating regular monitoring [21,39].

In many cases, a surgical approach may be required. In contrast to hormonal treatment, surgery is a definitive treatment of bladder endometriosis [30]. Another benefit is that the surgery allows for the simultaneous excision of endometriosis lesions from other locations [30]. Before the surgery, a precise diagnosis for evaluating the size and localization of the endometriosis nodule, as well as hydronephrosis, is mandatory. For this purpose, we used transvaginal and kidney sonography and cystoscopy. Recent guidelines recommend ultrasound as the first-line tool in deep infiltrating endometriosis, while cystoscopy is considered a useful method for the precise evaluation of the bladder nodule [37,45]. Depending on this information, an interdisciplinary procedure with a urologist may be required. The goal should be a complete resection of the endometriosis nodule while also preserving healthy tissue, which, on the one hand, facilitates significant improvements in pain and urinary symptoms and, on the other, minimizes the recurrence risk [46,47,48]. Using D-J stents for the surgery is optional. To the best of our knowledge, there are no studies approving the usefulness of D-J stents for the surgery. In our recent publication, we detailed the outcomes of D-J stents in patients undergoing laparoscopy due to deep endometriosis. We compared three groups: those without D-J stents, those with D-J stents removed immediately during the surgery, and those with D-J stents remaining for at least two weeks [49]. Another group investigated the advantages of D-J in patients with deep endometriosis, even with ureteral involvement [25]. Both studies reported no difference in terms of ureter injury in patients with and without D-J stents. The risk of bladder infection, on the other hand, is significantly higher in patients with D-J stents [25,49]. We used to use D-J stents standardly in surgeries for deep endometriosis. However, with growing experience, we started using them only in selected cases. For example, in cases where an endometriosis nodule is located in the trigone or in close proximity to the ureters (less than 2 cm), we take precautions to prevent potential ureter obstruction caused by postoperative bladder tissue swelling. In another scenario, if the nodule is large, measuring more than 3–4 cm, an enhanced bladder reconstruction should be anticipated. Or, in the case of hydronephrosis, discharging the kidney should be anticipated. In the present study, we compared patients with and without D-J and found no difference (Table 3).

There are several options for the surgical treatment of bladder endometriosis, such as transurethral resection, laparoscopic partial bladder resection, and shaving [21,29]. However, certain factors make transurethral resection an ineffective treatment for bladder endometriosis [25,26,27,28]. Since the endometriosis lesion grows from the outer layer to the inner layer, a complete resection seems unachievable. Furthermore, in many cases, the bladder mucosa is not affected and the nodule cannot be detected by cystoscopy. On the one hand, this method carries a high risk of bladder perforation, and on the other hand, it also carries a high risk of recurrence [25,26,27,28]. For these reasons, we do not recommend this option as a sole treatment for bladder endometriosis. On the other hand, reports indicate that a bladder part resection is an effective method that yields good long-term results in terms of symptoms and carries a low recurrence risk [21,36]. To lower the chance of recurrence, the excision must be complete, leaving about 2 mm of healthy tissue around the nodule. Surgeons should also control the mucosa by looking at it and touching it to make sure that there is no visible lesion in the mucosa and no palpable nodularity of the bladder wall [30]. The extent of dissection directly depends on the localization and size of the endometriosis nodule. For example, nodules can be in the front wall of the bladder, or big nodules that need to be cut out of the bladder wall may need to be accessed through the space of Retzius and moved up to the level of the urachus. In contrast, lesions on the dome may require minimal mobilization. Nodules of the bladder base/posterior wall or trigone require entry into the uterovesical space [30]. If the lateral bladder wall is affected, it may be necessary to dissect the paravesical, paravaginal, and pararectal spaces. We have recently published a video article on lateral wall endometriosis [50]. The crucial point was that the bladder dome and peritoneum were unaltered (Figure 3C). An enhanced dissection was required for reaching the endometriosis nodule. For this reason, surgeons must be familiar with the avascular spaces and anatomy of the pelvis [51]. In our case series, 27 patients underwent laparoscopic partial bladder resection. The average nodule size was 2.43 cm. Many authors asserted that gynecologists can perform a partial bladder resection if the distance between the endometriosis nodule and ureteral orifices is at least 2 cm. The authors of this study are in line with this statement. In our study, the involvement of a urologist was necessary in four cases. Three patients experienced ureteral obliteration due to endometriosis, which required the preparation of a ureteroneocystostomy. In one case, the distance between the nodule and ureteral orifices was less than 2 cm. Postoperative patients should obtain a bladder catheter for 10 to 14 days [30]. Rich bladder vascularization and sterile content enable a favorable healing process [52]. Many authors considered a bladder part resection as a safe method with a low complication risk [26,36,47,48]. The most common complications are bleeding and leaking [30].

Another surgical option is shaving. Many authors reported favorable postoperative results after shaving and low complication risk [15,53,54,55]. In the present study, ten patients underwent shaving. In all cases, the cystoscopy showed no mucosa infiltration. In six cases, we performed bladder suture after deep shaving to prevent a fistula. There were neither intraoperative nor postoperative complications. This method has some advantages, such as shorter hospital stays and shorter postoperative bladder catheterization [56]. Both methods have been shown to significantly improve symptoms. In the presented case series, improvements in dysuria and symptoms, in general, were 100% and 90%, respectively.

We only used resorbable suture for the suture (Table 2). We used polyglactin suture in 59.5% of cases, barbed suture in 12%, and PDS in 7% for bladder sutures. Our analysis revealed no variation in the suture time, postoperative complications related to bleeding and bladder dysfunction, or symptom improvement. Several authors have used or suggested the aforementioned suture materials [29,57].

We debate whether urologists, gynecologists, or urogynecologists should perform this surgery. From our point of view, it depends on the local rules of the hospital and the location and size of the endometriosis nodule. In many cases, a gynecologist may carry out the surgery, since the nodule lies mostly in the bladder dome. However, excellent laparoscopic surgical skills and mastery of suture technique, as well as excellent knowledge of pelvic anatomy, bladder structure, and avascular spaces, are required. In particular cases (e.g., if the ureter is affected, the location of the nodule is unfavorable, or the size is big), the surgery should be performed interdisciplinarily with a urologist.

The risk of endometriosis-associated malignancies of the urinary tract is very low. The literature has reported only a few cases of endometriosis-associated malignancies in the urinary tract, most of which involved the bladder [58].

## 5. Conclusions

This analysis demonstrated that gynecologists could perform laparoscopic surgical treatment of bladder endometriosis. However, we recommend an intervention by an interdisciplinary team, including a gynecologist and a urologist, if a ureteroneocystostomy is required or the localization of an endometriosis nodule is unfavorable. Laparoscopic partial bladder resection and shaving seem to be appropriate methods for improving urinary symptoms, with a low rate of intra- and postoperative complications even in patients with large endometriosis nodules. However, it is important to consider potential complications like bleeding, pollakiuria, bladder dysfunction, incomplete bladder healing, or suture leak. This surgical approach requires excellent laparoscopic skills.

## Figures and Tables

**Table 1:** Patient characteristics.

# of patients	42
Age (mean ± SD)	33.98 ± 6.32
BMI kg/m^2^ (mean ± SD)	24.62 ± 7.24
Symptoms:	
Dysmenorrhea	37 patients (88%)
VAS* score of dysmenorrhea	(mean ± SD VAS* = 7 ± 0.9)
Dysuria	29 patients (69%)
VAS* score of dysuria	(mean ± SD VAS* = 7.1 ± 1.9)
Dyspareunia	16 patients (38%)
VAS* score of dyspareuina	(mean ± SD VAS* = 5.4 ± 1.2)
Dyschezia	10 patients (24%)
VAS* score of dyshezia	(mean ± SD VAS* = 4.1 ± 10.9)
Lower abdominal pain	5 patients (12%)
Hematuria	4 patients (9.5%)
Birth in history	7 patients (17%)
Cesarean section	3 patients (7%)
Vaginal delivery	4 patients (9.5%)
Surgery in history	25 patients (59.5%)–30 laparoscopies, 3 cesarean section, 6 curettages in total
Laparoscopy:	21 patients (50%)
1× Laparoscopy	16 patients (38%)
2× Laparoscopies	3 patients (7%)
≥3× Laparoscopies	2 patients (5%)
Endometriosis laparoscopy	20 patients (47.5%)–23 laparoscopies
Cesarean section	3 patients (5%)
Hysterectomy	2 patients (5%)

* VAS—Visual Analog Scale. The severity of the symptoms is presented as an average value.

**Table 2:** Intraoperative outcome.

Total Number	42 Patients (100%)
Bladder resection:	37 patients (88%)
Partial cystectomy	27 patients (64%)
Shaving	10 patients (24%)
No bladder resection	5 patients (12%)
Size of endometriosis nodule cm	
Average (mean ± SD)	2.43 ± 0.97
<2 cm	13 patients (31%)
2–4 cm	25 patients (59.5%)
>4 cm	1 patient (2.5%)
n.a.	3 patients (7%)
D-J Stent	16 patients (38%)
Thread	33 patients (78.5%)
Vicryl (polyglactin)	25 patients (59.5%)
Barbed (V-Loc)	5 patients (12%)
PDS	3 patients (7%)
Adenomyose	27 patients (64%)
Adhesion Uterus/Bladder	29 patients (69%)
Cystoscopy	
Detected	25 patients (59.5%)
Not detected	17 patients (40.5%)
Involvement of urologist	3 patients (7%)
Ureteroneocystostomy	2 patients (5%)
Close to trigone *	5 patients (12%)

* the distance between the nodule and trigone is less than 2 cm.

**Table 3:** Comparison of two groups, with and without D-J stents.

Groups	With D-J	Without D-J	*p* Value
# of patients	16 patients	21 patients	
Age (mean ± SD)	33.63 ± 5.83	34.43 ± 6.84	0.7098
BMI kg/m^2^ (mean ± SD)	24.07 ± 4.89	23.75 ± 8.36	0.8925
Symptoms:			
Dysmenorrhea	14 patients	21 patients	0.1802
Dysuria	11 patients	14 patients	1.0000
Dyspareunia	7 patients	6 patients	0.4891
Dyschezia	5 patients	3 patients	0.2540
Lower abdominal pain	1 patient	4 patients	0.3641
Hematuria	1 patient	2 patients	1.0000
Size of the endometriosis nodule			
Average size cm	2.63 ± 1.15	2.26 ± 0.85	0.2676
<2 cm	5 patients	6 patients	
2–4 cm	9 patients	14 patients	
>4 cm	1 patient	0 patient	
Nodule close to trigone	2 patients	1 patient	0.5676
Adhesions Uterus/Bladder	12 patients	16 patients	1.0000
Bladder part resection	13 patients	13 patients	0.2847
Shaving	3 patients	7 patients	0.4613
Involvement of urologist	4 patients (ureteroneocystostomy)	0 patients	0.0276
Improvement of symptoms	11 patients	15 patients	1.0000
Complication	2 patients	4 patients	0.6796
Bleeding	0 patients	3 patients	0.2432
Ureteral stenosis	0 patients	1 patient	1.0000
Pollakiuria	1 patient	0 patients	0.4324
Bladder dysfunction	1 patient	0 patients	0.4324

## Data Availability

The raw data supporting the conclusions of this article will be made available by the authors on request.
